# A single oral dose of an iso-alpha acids rich hop extract dampens the lipoteichoic acid mediated immune response of monocytes in healthy individuals

**DOI:** 10.1007/s00394-026-03931-x

**Published:** 2026-03-07

**Authors:** K. Csarmann, F. Jung, A. Baumann, B. Simbrunner, K. Schweiger, K. Burger, R. Staltner, C. Hellerbrand, I. Bergheim

**Affiliations:** 1 Department of Nutritional Sciences, Molecular Nutritional Science, University of Vienna, Josef-Holaubek-Platz 2 (UZA II), 1090 Wien Vienna, Austria; 2 Vienna Doctoral School of Pharmaceutical, Nutritional and Sport Sciences, University of Vienna, Vienna, Austria; 3 Division of Gastroenterology and Hepatology, Department of Internal Medicine III, Medical University of Vienna, Vienna, Austria; 4 Institute of Biochemistry, Friedrich-Alexander-Universität Erlangen-Nürnberg, Erlangen, Germany

**Keywords:** Iso-alpha acids, Lipoteichoic acid, Monocytes, Toll-like receptor 2, Cytokines

## Abstract

**Purpose:**

Bacterial infections significantly contribute to global mortality. Lipoteichoic acid (LTA), a key component of Gram-positive bacterial cell walls, triggers immune responses via Toll-like receptor 2 (TLR2). Iso-alpha acids (IAA), bitter compounds derived from hops (*Humulus lupulus L.)*, are known for their anti-inflammatory properties, but their effects on human immune modulation remain unclear. This study explored the effect of a single oral dose of IAA on LTA-induced inflammatory responses in human immune cells and underlying molecular mechanisms.

**Methods:**

In a pilot study in healthy female volunteers (*n* = 5) dose- and time-dependent effects (0–90 mg IAA) on LTA-induced immune responses in monocytes were assessed. A randomized, placebo-controlled cross-over study (*n* = 13, male and female) evaluated the acute effects of 15 mg IAA. Peripheral blood mononuclear cells (PBMCs) were stimulated with LTA ex vivo, and cytokine release (IL-6, IL-1β) was measured. Mechanistic studies using J774A.1 cells and TLR2-transfected HEK293 cells explored the molecular pathways of IAA.

**Results:**

In the pilot study, 15 mg IAA was identified as the most tolerated dose in terms of taste for the participants which still had a marked effect on LTA-mediated IL-6 release from monocytes. In the main study, PBMCs from IAA-treated participants showed significantly lower IL-6 and IL-1β secretion after LTA stimulation compared to placebo (*p* < 0.05). In vitro, IAA suppressed JNK-mediated inflammatory signaling without affecting TLR2 activation.

**Conclusion:**

A single low-dose intake of IAA from hops reduces LTA-induced cytokine release in blood immune cells, likely via JNK inhibition, suggesting its potential role in modulating inflammatory responses.

**Graphical abstract:**

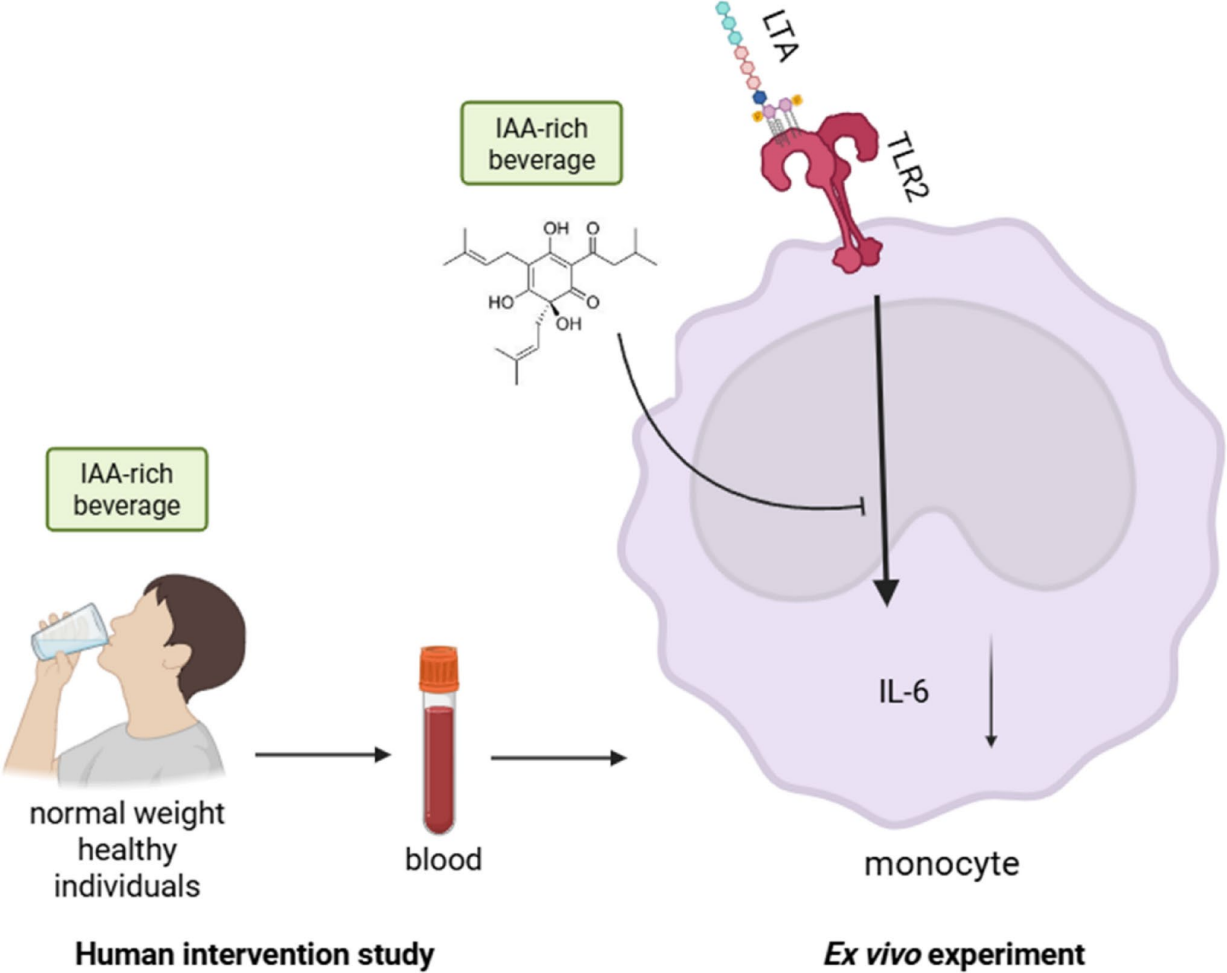

**Supplementary Information:**

The online version contains supplementary material available at 10.1007/s00394-026-03931-x.

## Introduction

Bacterial infections still cause a significant number of deaths worldwide every year [1], and antibacterial resistance (AMR) marks a continuous challenge in the management of infections. For instance, it has been estimated that in 2021 ~ 1.14 million deaths worldwide were attributable to bacterial AMR [2]. Results of the Global Burden Disease 2021 Antimicrobial Resistance Collaborators suggest that AMR deaths in children under the age of 5 years decreased in the last decades [2], whereas the number of deaths related to AMR in those older than 70 years being among the fastest growing population world-wide, is steadily increasing [2]. Gram-positive bacteria like *Staphylococcus aureus*, *Streptococcus pneumoniae* and a variety of bacteria summarized under the so-called vancomycin-resistant *Enterococci*, have been shown to have a highly variable growth and resistance pattern. These bacteria are also among the leading bacterial cause of death in over 15-year-old individuals in 135 countries [1, 3]. Studies suggest that not only the bacteria and their growth but also surface structures which in the case of Gram-positive bacteria include lipoproteins, peptidoglycans, poly-N-acetyl-glucosamine, wall teichoic acid and lipoteichoic acid (LTA) are critical in the inflammatory response related to bacteria [4–6]. LTA is indispensable in *Staphylococcus aureus* [7] and has been shown to induce inflammatory responses in the host through Toll-like receptor 2 (TLR2), a pattern recognition receptor (PRR) that either forms homodimers or heterodimers with TLR1 or TLR6 [8]. Previous studies suggest that similar to the recognition of bacterial endotoxin by TLR4 [9, 10], CD14 can bind LTA and transfer the bacterial wall compound to TLR2 [11, 12] subsequently leading to an induction of proinflammatory signaling cascades and a release of respective cytokines [13–15].

For many centuries hop (*Humulus lupulus L.*) has been used in beer brewing and in traditional medicine for the treatment of insomnia but also inflammatory diseases [16, 17]. Indeed, hop constitutes are added to the brewing process for their taste but also their antimicrobial properties [18]. In vitro studies have suggested that bitter acids like alpha-acids such as humulone found in hop possess antimicrobial properties against Gram-positive bacteria including *Staphylococcus*, *Clostridium* and *Bacillus* [19–23]. Moreover, results of animal studies suggest that the oral intake of iso-alpha acids (IAA) derived from alpha acids upon thermal isomerization [24] can attenuate inflammatory responses like the induction of proinflammatory cytokines or oxidative stress in liver tissue and brain in models of alcohol- and diet-related liver damage and Alzheimer’s disease [13, 25, 26]. Results of in vitro studies suggest that IAA may alter NFκB and AP-1 activation through interfering with JNK and IKKbeta activation [25, 27]. Pharmacokinetic studies in humans have shown that an intake of ~ 600–800 ml of commercially available high- and low hopped beer (Pale Ale: ~40 mg/l and wheat beer: ~10 mg/l IAA) leads to blood trans-IAA concentrations of ~ 0.1 and 0.02 mg/l, respectively, peaking at 0.5 h and dropping to ~ 0.01–0.02 mg/l and 0.003 mg/l after 2 h [28]. It has also been reported that IAA was measurable up to 6 h after consumption. These data suggest that IAA is absorbed, bioavailable and may exert its physiological effects over a prolonged period after ingestion [28, 29]. If iso-alpha acids when consumed in doses corresponding to those found when consuming moderate amounts of alcohol-free or alcohol containing beer, have effects on the immune response in humans has not yet been clarified.

Starting from this background, the aim of this study was first to determine in a pilot study if the oral intake of low doses of an IAA-rich hop extract (0–90 mg IAA) at concentrations also reached in the brewing process of alcohol containing or alcohol-free beer is related with alterations of the immune response of blood derived monocytes and how long these effects are prevalent in young healthy women. In a second randomized placebo-controlled crossover study, we assessed the effect of a onetime ingestion of an IAA-rich hop extract (15 mg IAA) or placebo in a mixed-sex cohort of young healthy individuals and determined related molecular mechanisms.

## Materials and methods

### Human intervention studies

All intervention studies were carried out in a randomized placebo-controlled cross-over design and in accordance with the ethical standards laid down in the Declaration of Helsinki of 1975 as revised in 1983. Studies were approved by the ethics committee of the University of Vienna, Vienna, Austria (reference numbers: 00367 and 00848) and were registered at clinical trials (NCT04847193, 16.03.2021; NCT06286644, 17.01.2024). The following exclusion criteria were defined for both studies: (1) following a special diet or (2) food malabsorption or (3) a history of diseases of the gastrointestinal tract or (4) the use of anti-inflammatory medication, whereas the following inclusion criteria were defined: normal weight (BMI > 18.5 kg/m^2^ or < 24.9 kg/m^2^), non-smoking people were enrolled after giving written informed consent and they should not have a viral or bacterial infection 3 weeks prior the study. In both studies, the participants were asked to refrain from hop-containing products two weeks prior to the studies and during the intervention.

#### Pilot study to determine time- and dose-response

**Fig. 1:**
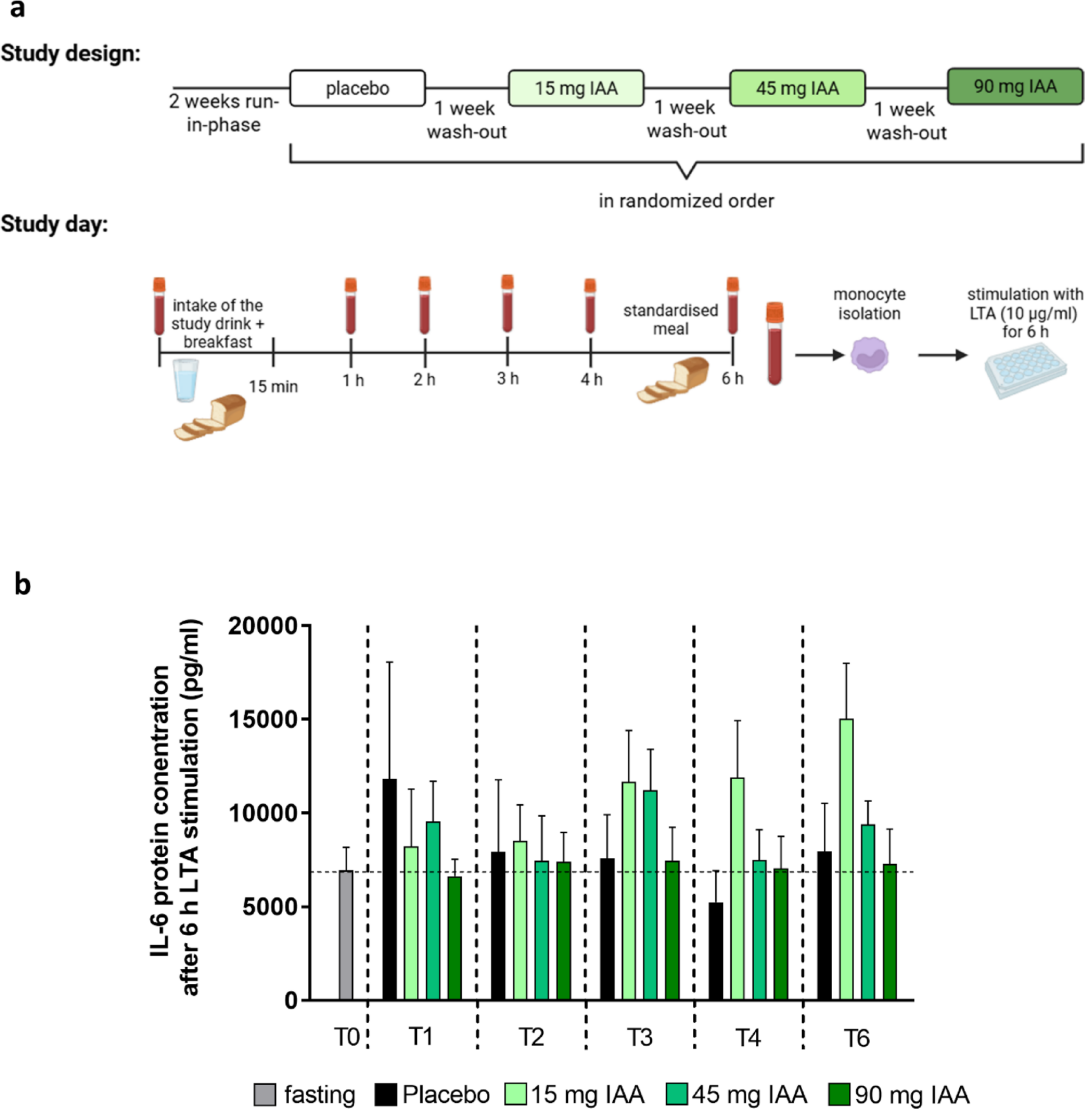
Effect of a single intake of different concentrations of an iso-alpha acids rich hop extract on the LTA-induced immune response of IL-6 protein concentration in monocytes of healthy study participants over time. **a** Study design and study day of the time- and dose-response study, **b** protein concentration of IL-6 in cell culture supernatant of monocytes stimulated with 10 µg/ml LTA for 6 h isolated from healthy female study participants of the time- and dose-response study receiving either a placebo or IAA rich hop extract in different concentrations (0 mg, 15 mg, 45 mg or 90 mg). Data are presented as means ± SEM, *n* = 5. The mean of IL-6 protein concentration of LTA-stimulated monocytes from fasting blood (T0) of all intervention groups are shown in one column. IAA: iso-alpha acids, IL: interleukin, LTA: lipoteichoic acid

To determine the optimal time of cell isolation after the ingestion of the IAA-rich hop extract and the optimal IAA doses, a single blinded, randomized pilot study was conducted in normal weight, healthy female volunteers. To prepare the study drink, which was prepared freshly right before the intake, 10 ml drinking water were mixed with thickener (Nestlé S.A., Vevey, Switzerland), sugar-free lemon flavour (SodaStream GmbH, Frankfurt am Main, Germany) and 0, 15, 45 or 90 mg IAA derived through an IAA-rich hop extract (Isohop^®^, generous gift from Barth-Haas Group GmbH & Co. KG Nuremberg, Germany). After an overnight fast and obtaining a blood sample, subjects were asked to consume the study drink within 15 min together with a standardised breakfast (2 medium-sized pretzels with 30 g butter). Blood was taken at 1, 2, 3, 4 and 6 h after consumption of the study drink. Between time point 4 and 6 there was another standardised meal. After the first study day and a 1-week wash-out phase, in which the participants again were asked to refrain from all hops containing foods and beverages, the intervention was repeated in a cross-over design until all four study days were completed. The study design and the procedure on the study day are summarized in Fig. 1a. Clinical parameters e.g. fasting glucose, aspartate aminotransferase (AST), alanine aminotransferase (ALT), and cholesterol were determined in the serum and monocytes were isolated from the whole blood as detailed below.

#### Assessment of the acute effects of the IAA-rich hop extract

**Fig. 2:**
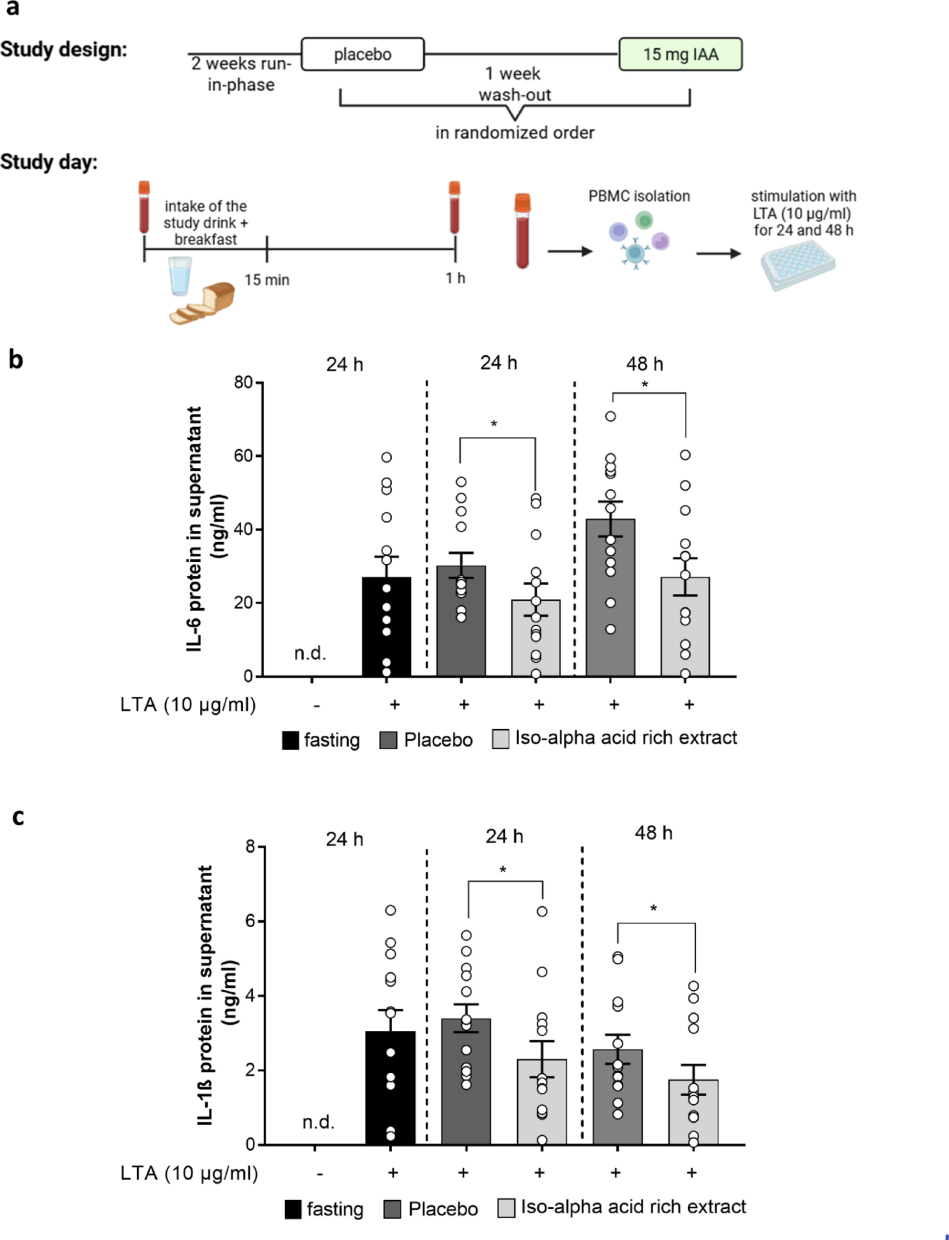
Effect of a single intake of 15 mg of an iso-alpha acids rich hop extract on the LTA-induced immune response of proinflammatory cytokines in PBMCs of healthy study participants. **a** Study design and study day of the assessment of the acute effects of IAA, protein concentrations of (**b**) IL-6 and (**c**) IL-1ß in cell culture supernatant of PBMCs stimulated with 0–10 µg/ml LTA for 24 h and 48 h isolated from healthy study participants of the assessment of the acute effects of IAA receiving either a placebo or 15 mg IAA rich hop extract. Data are presented as means ± SEM, *n* = 13. *p < 0.05. IAA: iso-alpha acids, IL: interleukin, LTA: lipoteichoic acid, n.d.: not detectable, PBMC: peripheral blood mononuclear cell

In a second study which was based on the results of the time- and dose-response study, the effect of 15 mg IAA derived through an IAA-rich hop extract compared to a placebo were assessed. Male and female participants were randomly assigned in a single-blinded design to receive a study drink as described before, containing 0 or 15 mg of IAA derived through an IAA-rich hop extract. The study drink was consumed within 15 min together with a standardised breakfast (2 medium-sized pretzels with 30 g butter). The participants were fasted overnight and blood samples were taken before the study drink was consumed and 1 h after consumption of the study drink. After the first study day and a 1-week wash-out phase, in which the participants were again asked to refrain from hops-containing foods and beverages, the intervention was repeated in a cross-over design. The study design and the procedure on the study day is summarized in Fig. 2a. Clinical parameters were determined in the serum and PBMCs were isolated from whole blood as detailed below. Demographic characteristics of the study subjects are reported in Tables 1 and 2.

**Table 1:** Characteristics of healthy female subjects enrolled in the dose- and time- response study

Parameter	Baseline
Sex (m/f)	0/5
Age (years)	26.4 ± 1.7
Body weight (kg)	56.1 ± 0.8
Height (m)	1.7 ± 0.0
BMI (kg/m^2^)	20.4 ± 0.7
Fasting glucose (mg/dl)	86.0 ± 3.4
AST (U/L)	22.6 ± 3.4
ALT (U/L)	17.0 ± 3.3
Cholesterol (mg/dl)	160.4 ± 9.8
Triglycerides (mg/dl)	65.6 ± 4.1

Values are means ± standard error of means. ALT: alanine aminotransferase; AST: aspartate aminotransferase; BMI: body mass index

**Table 2:** Characteristics of healthy subjects enrolled in the study to assess the acute effect of IAA on LTA-dependent immune response of PBMCs

Parameter	Baseline
Sex (m/f)	6/7
Age (years)	26.5 ± 0.9
Body weight (kg)	64.2 ± 2.1
Height (m)	1.7 ± 0.0
BMI (kg/m^2^)	21.7 ± 0.3
Fasting glucose (mg/dl)	88.2 ± 1.9
AST (U/L)	23.5 ± 1.6
ALT (U/L)	27.6 ± 3.8
Cholesterol (mg/dl)	165.8 ± 4.3
Triglycerides (mg/dl)	68.0 ± 7.4

Values are means ± standard error of means. ALT: alanine aminotransferase; AST: aspartate aminotransferase; BMI: body mass index; IAA: iso-alpha acids; LTA: lipoteichoic acid; PBMC: peripheral blood mononuclear cell

### Isolation of monocytes

Monocytes were isolated from whole blood samples collected from participants of the time- and dose-response study or from healthy, naïve, normal weight donors. After obtaining written informed consent and an overnight fast, blood was drawn from healthy, normal weight donors, with approval from the ethics committees of the University of Vienna, Vienna, Austria (reference number 00586). Monocytes were isolated from whole blood samples as detailed before using a density gradient centrifugation and the pluriSelect system following the instructions of the manufacturer (pluriSelect Life Science UG & Co. KG, Leipzig, Germany). In brief, blood was layered onto monocytes density gradient media (1.068 g/ml) in separation tubes (pluriSelect Life Science UG & Co. KG, Leipzig, Germany) and centrifuged at room temperature for 15 min. Cells were collected and used for further experiments as described below.

### Isolation of PBMCs

PBMCs were isolated from whole blood samples as described in detail before [11]. In brief, cells were isolated by density gradient centrifugation. In brief, blood was layered onto Pancoll solution (1.077 g/ml) and centrifugated for 30 min at room temperature. Cells were collected and used for further experiments as described below.

### Stimulation of monocytes and PBMCs obtained after an oral ingestion of iso-alpha acids derived through an IAA-rich hop extract

Isolated monocytes and PBMCs were cultivated in RPMI-1640 medium (Sigma-Aldrich Chemie GmbH, Steinheim, Germany) with 10% fetal bovine serum (Pan-Biotech GmbH, Aidenbach, Germany) and 100 µg/ml streptomycin and 100 U/ml penicillin (Pan-Biotech GmbH, Aidenbach, Germany) at 37 °C in a humidified 5% CO₂ atmosphere for 1 h. Subsequently, cells were either challenged with 0–10 µg/ml LTA for 6 h in the pilot study and for 24 and 48 h in the assessment of the acute effects study, respectively. Cell culture supernatant was collected at the end of the experiment and stored at − 80 °C until further use.

### Experiments in monocytes isolated from naïve healthy donors

Isolated monocytes were cultivated in RPMI-1640 medium (Sigma-Aldrich Chemie GmbH, Steinheim, Germany) with 10% fetal bovine serum (Pan-Biotech GmbH, Aidenbach, Germany) and 100 µg/ml streptomycin and 100 U/ml penicillin (Pan-Biotech GmbH, Aidenbach, Germany) at 37 °C in a humidified 5% CO₂ atmosphere for 1 h. Subsequently, cells were preincubated for 1 h with different concentrations of iso-alpha acids (0.3125–1.25 µg/ml) and then treated with 10 µg/ml LTA for 6 h. Cell culture supernatant was collected at the end of the experiment and stored at − 80 °C until further use.

### Cell culture experiments with hTLR2 transfected HEK cells

A commercially available reporter gene assay with TLR2 transfected HEK293 cells was used to assess the effects of iso-alpha acids on TLR2 ligand concentration (InvivoGen, Toulouse, France). The transfected human TLR2 cells were grown according to the instructions of the manufacturer. In brief, cells were grown in a humidified, 5% carbon dioxide atmosphere using DMEM media (Pan-Biotech GmbH, Aidenbach, Germany) containing 10% fetal bovine serum (Pan-Biotech GmbH, Aidenbach, Germany) and 100 µg/ml streptomycin and 100 U/ml penicillin (Pan-Biotech GmbH, Aidenbach, Germany). After reaching 80% confluence, cells were challenged with 0–10 µg/ml LTA in the presence of 0–40 µg/ml iso-alpha acids dissolved in HEK-Blue™ Detection medium (InvivoGen, Toulouse, France) for 12 h. Colour changes of the medium being indicative of ligand concentration and binding were determined at 655 nm.

### Cell culture experiments with J774A.1 cells

J774A.1 cells (DMSZ, Braunschweig, Germany) showing a morphology similar to macrophages/monocytes, were cultured in Dulbecco´s Modified Eagle Medium (Pan-Biotech GmbH, Aidenbach, Germany) supplemented with 10% fetal bovine serum (Pan-Biotech GmbH, Aidenbach Germany), 100 µg/ml streptomycin and 100 U/ml penicillin (Pan-Biotech GmbH, Aidenbach, Germany) at 37 °C in a humidified 5% CO₂ atmosphere. At 80% confluency, cells were preincubated for 1 h with or without 5 ng/ml anisomycin (Biomol GmbH, Hamburg, Germany), afterwards they were preincubated for 1 h with iso-alpha acids (0–25 µg/ml) and then treated with 10 µg/ml LTA for 6 and 24 h. The supernatant was collected at the end of the experiment and stored at − 80 °C until further use.

### Enzyme-linked immunosorbent assays (ELISA´s)

IL-6 and IL-1ß protein concentrations were analysed in cell culture supernatant of monocytes and PBMCs or J774A.1 cells using commercially available DuoSet^®^ ELISA Development Systems (R&D^®^, Minneapolis, USA) kits.

### Western blot analysis

J774A.1 cells were homogenized in RIPA buffer (20 mmol/l 3 [N-morpholino] propanesulfonic acid, 150 mmol/l NaCl, 1 mmol/l ethylenediaminetetraacetic acid, 1% Nonidet P-40, and 0.1% sodium dodecyl sulfate) containing protease and phosphatase inhibitor cocktails (Sigma-Aldrich Chemie GmbH, Steinheim, Germany) to obtain protein lysates. Protein lysates (5 µg/lane) were separated on 10% SDS-polyacrylamide gels and transferred to polyvinylidene difluoride membranes (Bio-Rad Laboratories, Hercules, CA, USA). Membranes were further incubated with specific primary antibodies (phosphorylated c-Jun N-terminal kinase (JNK) and total JNK; phosphorylated p38 MAPK and p38 MAPK; Cell Signaling Technology, MA, USA) and the respective secondary antibody (anti-rabbit IgG, HRP-linked; Cell Signaling Technology, MA, USA). To detect the protein bands, Clarity Western Enhanced Chemiluminescence Substrate (Bio-Rad Laboratories, Hercules, CA, USA) and ChemiDoc XRS System (Bio-Rad Laboratories, Hercules, CA, USA) were used. Densitometric analyses of detected bands were performed with ImageLab (Bio-Rad Laboratories, Hercules, CA, USA).

### Statistical analyses

Data are presented as means ± standard error of the means (SEMs). Grubb´s test was performed before statistical analysis to identify outliers. Homogeneity of variances was tested and data were log-transformed if data were not normal distributed or in case of inhomogeneity of variances before performing further statistical tests. A paired t-test was used to determine statistically significant differences between interventions and to analyse differences between C- and LTA-stimulated cells of Western Blot analysis. One-way ANOVA was used for all other comparisons (GraphPad Prism Software, CA, USA). A p-value < 0.05 was defined as threshold for statistical significance.

## Results

### Effects of an acute oral intake of an IAA-rich hop extract on the LTA-induced IL-6 release in monocytes: assessment of time- and dose-response in a pilot study

Characteristics of the 5 study participants enrolled and analysed in the pilot study assessing the time- and dose-effect of an oral intake of IAA on the LTA-induced release of IL-6 are summarized in Table 1. IL-6 was selected as suitable target cytokine as it has been shown before in cell culture experiments that its bacterial toxin induced expression can be suppressed by IAA [13]. As expected, the challenge of monocytes isolated before and 1 h after the intake of the placebo resulted in a manifest ~ 1.85-fold increase in IL-6 protein levels in cell supernatant (see Supplemental Fig. 1). A similar effect of LTA was also found in cells isolated 2, 3, 4, and 6 h after the intake of the placebo (see Supplemental Fig. 1). In contrast, when monocytes isolated 1 h after the intake of the study drinks containing 15, 45, or 90 mg of IAA were stimulated with LTA for 6 h, the relative increase of IL-6 protein in cell supernatant compared to the fasted state (T0) was lower than after the intake of the placebo (Placebo: ~ + 1.7-fold; 15 mg IAA: ~ + 1.18-fold; 45 mg IAA: ~ + 1.38-fold; 90 mg IAA: ~ 0.956-fold) (see Fig. 1b). When cells isolated 2 h after the intake of the different IAA containing drinks were stimulated with LTA for 6 h, IL-6 protein levels were still lower when subjects had consumed the drink containing 90 mg of IAA. In contrast, IL-6 protein levels were slightly higher or similar in cell supernatant of cells isolated 2 h after the intake of 15 or 45 mg of IAA when compared to placebo. Also, 4 h and 6 h after the intake of the different IAA-enriched drinks, IL-6 protein levels in cell culture supernatant were either higher than after placebo consumption, or at the level of placebo (see Fig. 1b). No statistical differences were found for IL-6 protein concentrations in LTA-stimulated monocytes when comparing data between the interventions per time point (see Fig. 1b).

As the taste of IAA was perceived more tolerable by subjects at lower levels and effects were almost similar 1 h after IAA was consumed regardless of doses consumed, 15 mg of IAA was selected for all further in vivo studies as suitable IAA concentration and cells were isolated 1 h after the intake in the further human intervention study.

### Effect of the oral intake of an IAA-rich hop extract on the LTA-induced cytokine release in healthy, normal weight men and women

To assess if similar effects as the ones found with respect to the LTA-induced release of IL-6 in monocytes are also found for other cytokines in men and women, we conducted a cross-over design study asking healthy women and men to consume the IAA-rich beverage (15 mg IAA derived through the IAA-rich hop extract) or a placebo in a randomized order with ingestions been separated by at least one week of washout. Characteristics of study participants are summarized in Table 2. Of the 17 subjects enrolled, 3 had to be excluded from the analysis as cells did not respond to the stimulation. Moreover, one subject discontinued the study after the first intervention. All of the participants were healthy and normal weight. Also, as cell yields were rather low an isolation of sufficient numbers of monocytes were not possible. Therefore, experiments were carried out with PBMCs.

Concentrations of IL-6 and IL-1β protein were below the level of detection in cell supernatant of most subjects when cells were stimulated with vehicle. Therefore, these data are only shown for fasting samples (see Fig. 2b and c). In contrast, the challenge of PBMCs isolated from fasted subject before ingesting the different study drinks with LTA for 24 h resulted in a robust significant increase of IL-6 protein and IL-1β protein levels in cell culture media (see Fig. 2b and c). In line with these findings, IL-6 and IL-1β protein levels in cell supernatant of cells isolated 1 h after the ingestion of the placebo were similarly increased. In contrast, both IL-6 and IL-1β protein levels in cell culture media of cells isolated 1 h after the intake of 15 mg IAA in the IAA-enriched study drink decreased significantly by ~ 31% and ~ 32% compared to controls (see Fig. 2b and c). In cell supernatant obtained from cells isolated 1 h after the intake of IAA and stimulated with LTA for 48 h, protein levels of IL-6 and IL-1ß were also significantly lower than in those obtained from cells isolated 1 h after the ingestion of placebo (placebo vs. IAA-rich drink: IL-6: ~ − 37% and IL1-β: ~ − 32%, *p* < 0.05 for both differences) (see Fig. 2b and c).

### Effect of the IAA-rich hop extract on the LTA-dependent induction of IL-6 and IL-1β release, as well as TLR2 and MAPKinase signaling in primary human monocytes and J774A.1 cells

**Fig. 3:**
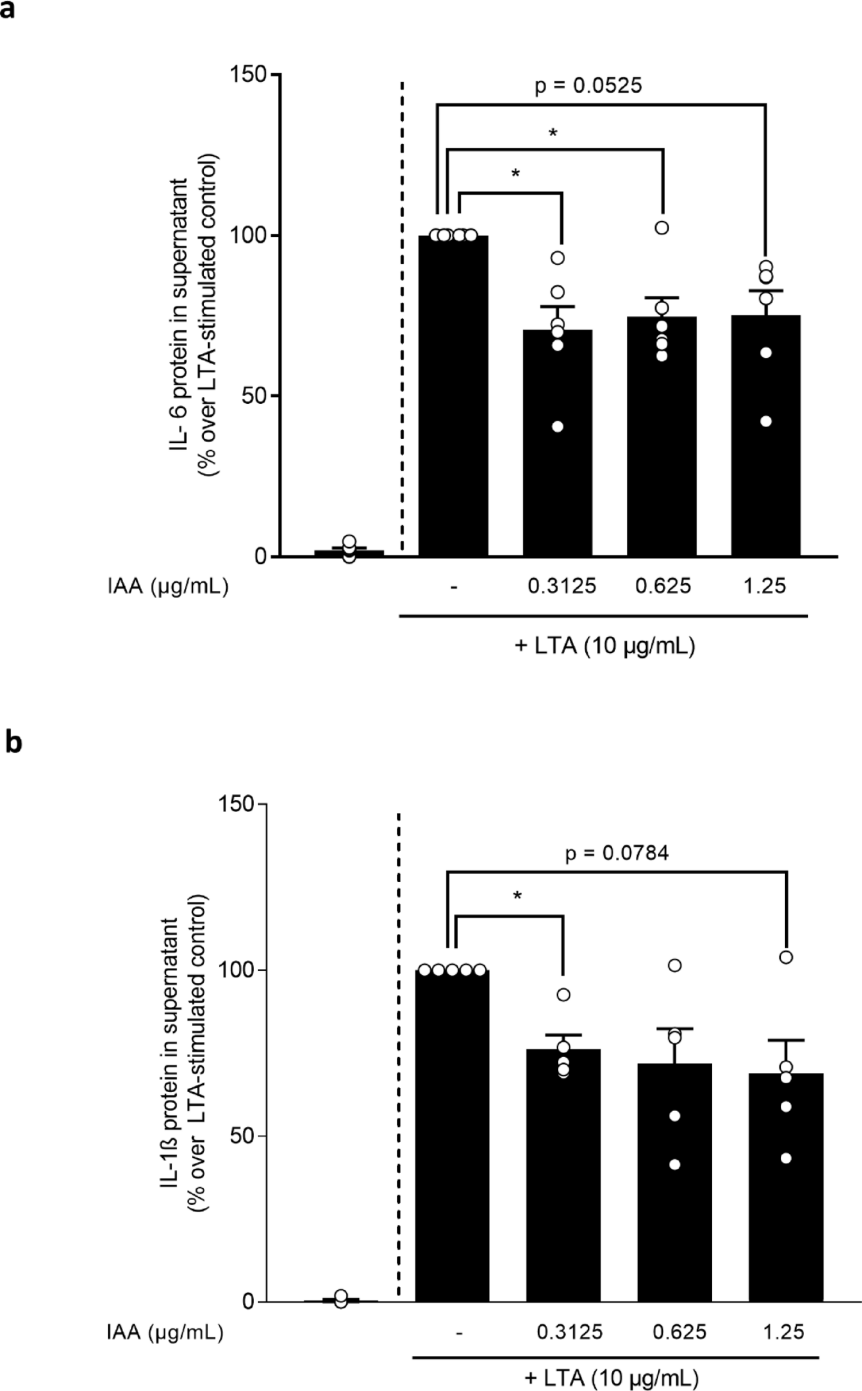
Effect of different concentrations of an iso-alpha acids rich hop extract on the LTA-dependent induction of IL-6 and IL-1ß protein concentration in monocytes. Protein concentration of (**a**) IL-6 and (**b**) IL-1ß in cell culture supernatant of monocytes isolated from healthy donors stimulated with 10 µg/ml LTA and different concentrations of IAA (0.3125–1.25 µg/ml) for 6 h. Data are presented as means ± SEM, *n* = 5–6. *p < 0.05. IAA: iso-alpha acids, IL: interleukin, LTA: lipoteichoic acid

To determine if the effects found in vivo were related to direct effects of the IAA-rich hop extract on monocytes, we next challenged monocytes isolated from healthy donors with LTA in the presence of different, low doses of the IAA-rich hop extract (0–1.25 µg/ml IAA). In line with the findings in vivo, the increase in IL-6 upon a 6 h long incubation with LTA was significantly attenuated when cells were pre-incubated for 1 h with 0.3125 µg/ml and 0.625 µg/ml IAA-rich hop extract before the challenge with LTA (see Fig. 3a). A similar trend was observed when cells challenged with LTA were concomitantly treated with 1.25 µg/ml IAA (*p* = 0.0525) (see Fig. 3a). The increase in IL-1ß protein concentration upon the challenge with LTA was significantly attenuated when cells were pre-treated for 1 h with 0.3125 µg/ml of IAA-rich hop extract (see Fig. 3b). A similar trend was observed when cells were co-treated with 1.25 µg/ml IAA-rich extract (*p* = 0.0784) (see Fig. 3b).

**Fig. 4:**
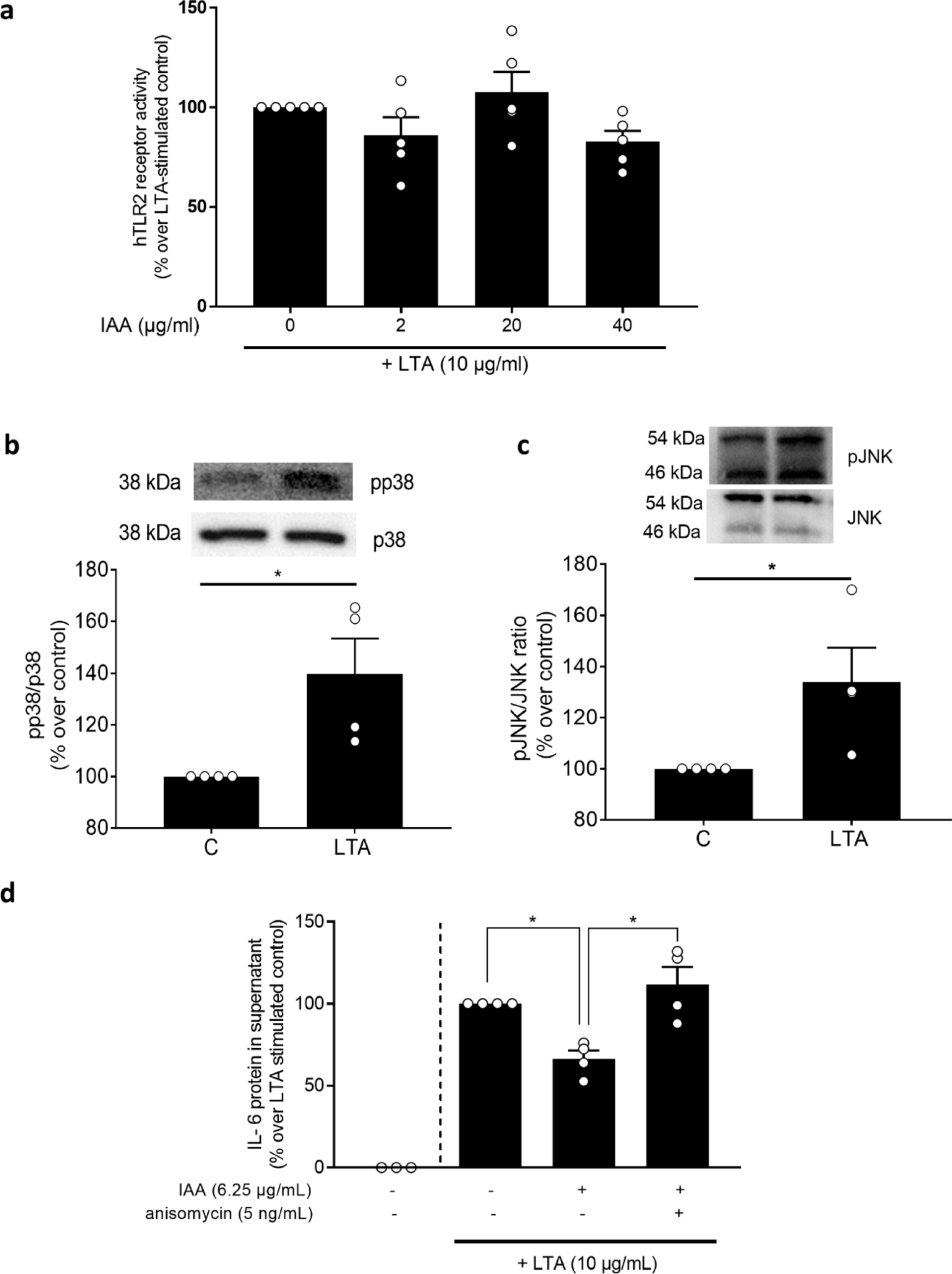
Effect of an iso-alpha acids rich hop extract on the LTA-dependent induction of TLR2 and MAPKinase signaling in J774A.1 cells. **a** hTLR2 receptor activity from HEK293 cells co-incubated with 10 µg/ml LTA and increasing concentrations of IAA (0–40 µg/ml) for 12 h, **b** phospho-p38/p38 protein in J774A.1 cell stimulated with 10 µg/ml LTA for 6 h, **c** phospho-JNK/JNK protein in J774A.1 cells stimulated with 10 µg/ml LTA for 6 h and **d** IL-6 protein concentration in cell culture supernatant of J774A.1 cells stimulated with 10 µg/ml LTA and 0–6.25 µg/ml IAA rich hop extract and 0 or 5 ng/ml anisomycin for 6 h. Data are presented as means ± SEM, *n* = 4–5. *p < 0.05. IAA: iso-alpha acids, IL: interleukin, JNK: c Jun N terminal kinase, LTA: lipoteichoic acid, hTLR2: human Toll-like receptor 2

As it has been suggested that some hop compounds (e.g., xanthohumol) dampen LTA-induced TLR2 signaling through interfering with the CD14-mediated transfer of the bacterial wall compound to TLR2 [11], we next determined if IAA also interfere with the LTA-dependent activation of TLR2 employing HEK-Blue™ TLR2 cell assay. Even when employing markedly higher concentrations than those used in vivo and shown to be effective in vitro in the present study, the preincubation of cells with IAA derived through the IAA-rich hop extract had no effect on the LTA-dependent activation of cells (see Fig. 4a).

As results of others suggest that IAA may attenuate the activation of p38 and JNK [27], we next challenged J774.A1 cells with LTA for 6 h which resulted in a significant increase in the phosphorylation of both, p38 (see Fig. 4b) and JNK (see Fig. 4c). Results of pilot studies revealed that the IAA-rich hop extract attenuated the increase in IL-6 protein in cell culture supernatant derived from J774.A1 cells upon a stimulation with LTA in an almost dose-dependent way (see Supplemental Fig. 2); however, doses of the IAA-rich extract needed were markedly higher (0–25 µg/ml). Based on these experiments, 6.25 µg/ml IAA derived through the hop extract was selected for all further experiments in J774.A1 cells (see Supplemental Fig. 2). The concomitant treatment of cells with 6.25 µg/ml IAA and 5 ng/ml anisomycin, being a JNK and p38 agonist [30], when being challenged with LTA, attenuated the protective effects of IAA significantly (see Fig. 4d).

## Discussion

### The oral intake of low doses of IAA derived through an IAA-rich hop extract attenuated the LTA-dependent stimulation of PBMCs and monocytes ex vivo

Despite the availability of a large number of antibiotics, Gram-positive bacteria still pose a significant risk for bacterial infections in humans even in highly developed countries, causing a wide range of diseases such as skin infections, pneumonia, endocarditis, urinary tract infections, and life-threatening bloodstream infections [3, 31]. Indeed, in recent years the antibiotic resistance is becoming an increasing concern [32], often limiting treatment options and subsequently leading to complications and even death. Results of in vitro studies indicated that IAA derived from hop may dampen LPS-dependent activation of immune cells [13] but may also have antibacterial effects. It has been shown in vitro that IAA has ionophores properties disrupting cell membranes thereby leading, especially in Gram-positive bacteria, to cell lysis [33]. Epidemiological evidence supporting a protective effect of IAA consumption against Gram-positive infection, including infections caused by *Staphylococcus aureus*, *Streptococcus pneumoniae*, or *Enterococcus spp*., is to our knowledge still lacking. In the present study, we determined the impact of an oral intake of `nutritionally´ relevant doses of IAA on the LTA-induced release of proinflammatory interleukins (e.g., IL-6 and IL-1β) in primary monocytes and PBMCs isolated from young healthy adults. Concentrations of IAA used in present studies were based on those found in 250–1000 ml of commercially available Porter or Lager beer [34]. In the brewing process, alpha acids often also referred to as humulones, are isomerized to iso-alpha acids or isohumulones tasting rather bitter. In the present study, we used a standardized solution of iso-alpha acids (30% w/w) produced from CO_2_ hop extract which when used in beer brewing is added to the beer after fermentation and before final filtration. While using lower doses than in most other studies in model organisms and cell cultures [35], we found that the LTA-dependent induction of the release of IL-6 protein from monocytes isolated from healthy women was markedly attenuated 1–2 h after the intake of the different IAA-rich drinks. Interestingly, results of our pilot data suggest that 1 h after the intake of all three drinks containing IAA effects were rather similar with respect to suppressing the release of IL-6 protein. This effect was lost when monocytes isolated 2 h after the intake of drinks containing 15 and 45 mg of IAA were stimulated with LTA while being persisted after the intake of the drink containing 90 mg of IAA. It has been shown before that IAA levels in blood increase as early as 30 min after the intake of beer but decline within 6 h back to baseline suggesting that IAA is rapidly taken up and metabolized [36, 37]. While not having been fully clarified yet, it has been suggested that iso-alpha acids are metabolized through phase 1 (cytochrome P450) and 2 enzymes [38]. Our results are in line with results of previous studies of us and others showing in in vitro experiments and experiments in animals a more prolonged suppression of the release of proinflammatory cytokines when employing higher doses of IAA [13, 25, 39]. In contrast to other studies, in the present study, we employed drinks to mimic the “nutrition” situation with dissolved IAA rather than capsulated IAA which would have been closer to a “pharmacological” setting. Still our approach was accompanied with the problem that IAA, which tastes rather bitter when consumed at higher concentrations, was fully tastable. Therefore, 15 mg were selected as a more suitable concentration of IAA to test effects in wider setting. In line with the findings of the pilot study, the oral intake of 15 mg of IAA derived through the IAA-rich hop extract suppressed the LTA-induced release of IL-6 and IL-1β from isolated PBMCs. Taken together, these data suggest that the intake of low doses of IAA may decrease the immune response of blood immune cells to LTA in humans for 1–2 h after the intake. As in the present study cells were exposed to the bacterial toxin ex vivo, further studies are needed to assess if similar effect are also found when humans suffer from an infection with Gram-positive bacteria. Also, it remains to be determined, if effects found are persistent when IAA are consumed repetitively.

### The effects of the IAA-rich extract on the LTA-dependent release of IL-6 are related to a suppression of an activation of JNK

Results of in vitro studies and animal models suggest that IAA may have direct effects on the inflammatory response of cells. Indeed, it has been shown before that in cell lines, like J774A.1 and L929sA, the pre-incubation with IAA before a challenge with bacterial endotoxin attenuates the release of IL-6 but also other proinflammatory mediators like RANTES and TNFα [13, 39] These data suggest that the immunosuppressive effects of IAA could attenuate an excessive and uncontrolled immune response during bacterial infections. Further studies are needed to determine if the immune suppressive effects found in the present study and in vitro and in vivo studies of others translate into e.g., a shortening of the time being infected or the severity of an infection caused by bacteria in humans. Interestingly in these studies, concentrations of IAA used were mostly markedly higher than in the present study needed in primary cells obtained from healthy humans (+ 20-40-fold compared to the present study, for overview see [13]). Somewhat in line with the findings of others, in the present study, experiments carried out in J774A.1 cells also required markedly higher doses of IAA when compared to those showing effects in primary human monocytes. Indeed, it has been suggested before that while affecting similar signaling pathways, (murine) cells line might require other (higher) doses of compounds when compared to primary cells [40–42]. Also, it has been shown, that this might be related to the species the cell lines were obtained from [43]. The protective effects of IAA with respect to inflammation have been related to an inhibition of the activation of NFκB but also agonistic effect on PPARα and γ (for overview see [35]). Moreover, it has been shown that MAPK and especially p38 (as well as JNK) are critical in the induction of IL-6 upon a challenge of cells with Gram-positive bacterial toxins such as LTA [44–46]. In the present study, the recognition of the Gram-positive bacterial toxin LTA by TLR2 was not altered as determined using stabily TLR2 transfected HEK cells. The challenge of J774A.1 cells challenged with LTA for 6 h showed a massive activation of both p38 and JNK phosphorylation. Also, when treating cells with anisomycin, a p38 and JNK activator [30], the protective effects of IAA were abolished. These results somewhat contrast those of others showing no effect of rho iso-alpha acids on p38 and JNK phosphorylation in LPS-stimulated RAW 264.7 cells [47]. However, it could be that differences are related to the challenge e.g., the use of LTA in the present study and LPS in the study of others as well as the use of IAA vs. rho iso-alpha acids. In summary, our data suggest that IAA even at low doses can attenuated the LTA-dependent induction of IL-6 and IL-1β protein release in primary human monocytes. Also, the protective effects of IAA seem to be related to altering JNK-/p38-dependent signaling. Further studies are needed to determine if IAA has direct effects of JNK and p38 or if indirect effects are involved.

### Limitations

Our study is not without limitations that need to be considered when interpreting the data. Specifically, while mostly employing primary human PBMCs and monocytes, all cell stimulation experiments were carried out ex vivo. Therefore, it remains to be determined if IAA, be they consumed as isolates or in an IAA-rich hop extract (30% w/w) as it was done in the present study, also affect the course of Gram-positive infections in vivo e.g., the length and the severity. Moreover, in the present study only acute effects resulting from a one-time intake of the IAA-rich extract were assessed. It remains to be determined if suppressing effects found with respect to alterations of bacterial toxin-dependent release of proinflammatory cytokines like IL-6 and IL-1β are also found when IAA are regularly consumed. Moreover, as no other bitter component was used as a control in the placebo drink, and the bitter taste of the IAA-rich extract is difficult to mask, it cannot to be ruled out that subjects were able to distinguish not only the placebo from the IAA-enriched drinks but also the different IAA-containing drinks. Also, in the present study, we only assessed effects in healthy, normal weight humans. It remains to be determined if effects alike are also found in overweight and/ or older individuals. Moreover, in the present study, we used an IAA-rich hop extract which besides IAA contains other hop compounds that may have affected the outcome. However, at the time of study, no pure IAA suitable for the use in human nutrition was available.

## Conclusion

Taken together, results of the present study suggest that an oral intake of IAA in low “nutritionally” relevant doses through an IAA-rich hop extract diminishes the LTA-dependent release of IL-6 and to a lesser extend also of IL-1β from monocytes in humans ex vivo. Results of our study also suggest that the immune suppressing effects with respect to the release of IL-6 persist for ~ 1–2 h. Our results also suggest that these effects of IAA may be related to an inhibition of JNK and p38; however, further studies are needed to determine mechanisms underlying the interaction of IAA with stress-activated protein kinases. Also, it remains to be determined if an intake of IAA affects Gram-positive bacterial infections in vivo and if this interaction improves disease outcome in humans.

## Supplementary Information

Below is the link to the electronic supplementary material.

Supplementary Material 1

Supplementary Material 2

## Data Availability

The original contributions presented in the study are included in the article/Supplementary Material, further inquiries can be directed to the corresponding author.

## Abbreviations

ALT: Alanine aminotransferase
AMR: Antimicrobial resistance
ANOVA: Analysis of variance
AP-1: Activator protein 1
AST: Aspartate aminotransferase
BMI: Body mass index
CD14: Cluster of differentiation 14
ELISA: Enzyme-linked immunosorbent assay
HEK: Human embryonic kidney (cells)
HRP: Horseradish peroxidase
IAA: Iso-alpha acids
IKKβ: IκB kinase beta
IL: Interleukin
JNK: c-Jun-N-terminal kinase
LTA: Lipoteichoic acid
MAPK: Mitogen-activated protein kinase
NFκB: Nuclear Factor kappa light chain enhancer of activated B cells
PBMC: Peripheral blood mononuclear cells
RIPA buffer: Radio-immunoprecipitation assay buffer
RPMI: Roswell park memorial institute (medium)
SEM: Standard error of the mean
TLR: Toll-like receptor
