# Supplementary material for: A single oral dose of an iso-alpha acids rich hop extract dampens the lipoteichoic acid mediated immune response of monocytes in healthy individuals
Source: Eur J Nutr. 2026 Mar 7;65(3):84. doi: 10.1007/s00394-026-03931-x (PMC12967542; doi:10.1007/s00394-026-03931-x)
Supplement: Supplementary file 1 — Supplementary Material 1 [file 394_2026_3931_MOESM1_ESM.pdf]

A single oral dose of an iso-alpha acids rich hop extract dampens the lipoteichoic acid mediated immune response of monocytes in healthy individuals

European Journal of Nutrition

Csarmann K, Jung F, Baumann A, Simbrunner B, Schweiger K, Burger K, Staltner R, Hellerbrand C, Bergheim I#

#**Corresponding author:** Ina Bergheim, Ph.D.
University of Vienna
Department of Nutritional Sciences
Molecular Nutritional Science
Josef-Holaubek-Platz 2 (UZA II)
A-1090 Wien
Phone: +43-1-4277-549 81
E-Mail: ina.bergheim@univie.ac.at

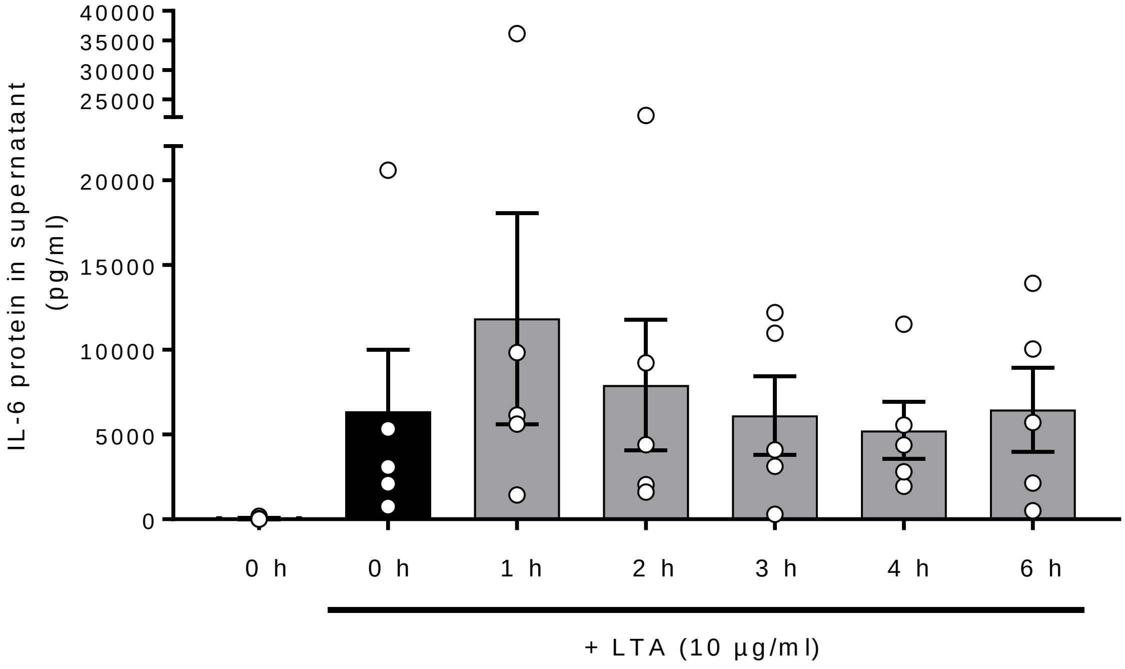

Supplemental Fig. 1 Effect of a 6 h incubation with LTA (10 µg/ml) on the IL-6 protein release in monocytes isolated from healthy women before and 1- 6 hours after the intake of the placebo Protein concentration of IL-6 in cell culture supernatant of monocytes stimulated with 10 µg/ml LTA for 6 h isolated from healthy female study participants of the time- and dose-response study receiving a placebo fasted and 1 – 6 h postprandial. Data are presented as means ± SEM, n = 5. IL: interleukin, LTA: lipoteichoic acid.
