# Supplementary material for: A single oral dose of an iso-alpha acids rich hop extract dampens the lipoteichoic acid mediated immune response of monocytes in healthy individuals
Source: Eur J Nutr. 2026 Mar 7;65(3):84. doi: 10.1007/s00394-026-03931-x (PMC12967542; doi:10.1007/s00394-026-03931-x)
Supplement: Supplementary file 2 — Supplementary Material 2 [file 394_2026_3931_MOESM2_ESM.pdf]

A single oral dose of an iso-alpha acids rich hop extract dampens the lipoteichoic acid mediated immune response of monocytes in healthy individuals

European Journal of Nutrition

Csarmann K, Jung F, Baumann A, Simbrunner B, Schweiger K, Burger K, Staltner R, Hellerbrand C, Bergheim I[#]

#**Corresponding author:** Ina Bergheim, Ph.D.
University of Vienna
Department of Nutritional Sciences
Molecular Nutritional Science
Josef-Holaubek-Platz 2 (UZA II)
A-1090 Wien
Phone: +43-1-4277-549 81
E-Mail: ina.bergheim@univie.ac.at

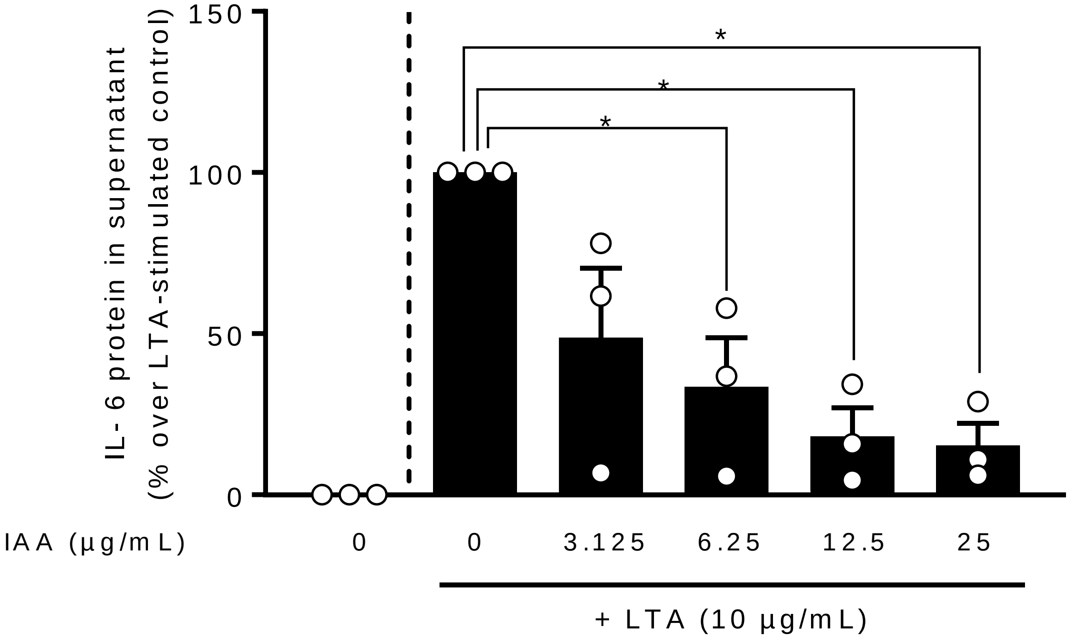

Supplemental Fig. 2 Effect of different concentrations of an iso-alpha acids rich hop extract on the LTA-dependent activation of J774.A1 cells Protein concentration of IL-6 in cell culture supernatant of J774A.1 cells stimulated with 0 or 10 µg/ml LTA and increasing concentrations of IAA rich hop extract (0 – 25 µg/ml) for 24 h. Data are presented as means ± SEM, n = 3. *p<0.05. IAA: iso-alpha acids, IL: interleukin, LTA: lipoteichoic acid.
